# An Overview of the Use and Applications of *Limosilactobacillus fermentum* in Broiler Chickens

**DOI:** 10.3390/microorganisms11081944

**Published:** 2023-07-29

**Authors:** Maria Paula Racines, Maria Nicole Solis, Miroslava Anna Šefcová, Róbert Herich, Marco Larrea-Álvarez, Viera Revajová

**Affiliations:** 1 Facultad de Ciencias Médicas Enrique Ortega Moreira, Carrera de Medicina, Universidad Espíritu Santo, Samborondón 092301, Ecuador; mracines@uees.edu.ec (M.P.R.); mnsolis@uees.edu.ec (M.N.S.); miroslava.sefcova@gmail.com (M.A.Š.); 2 Department of Morphological Disciplines, University of Veterinary Medicine and Pharmacy, 040 01 Košice, Slovakia; robert.herich@uvlf.sk

**Keywords:** *Limosilactobacillus fermentum*, broiler chicken, gut health, microbial diversity, immune response modulation

## Abstract

The implementation of government regulations on antibiotic use, along with the public’s concern for drug resistance, has strengthened interest in developing alternatives not only aimed at preserving animal production but also at reducing the effects of pathogenic infections. Probiotics, in particular, are considered microorganisms that induce health benefits in the host after consumption of adequate amounts; they have been established as a potential strategy for improving growth, especially by stimulating intestinal homeostasis. Probiotics are commonly associated with lactic acid bacteria, and *Limosilactobacillus fermentum* is a well-studied species recognized for its favorable characteristics, including adhesion to epithelial cells, production of antimicrobial compounds, and activation of receptors that prompt the transcription of immune-associated genes. Recently, this species has been used in animal production. Different studies have shown that the application of *L. fermentum* strains not only improves the intestinal ecosystem but also reduces the effects caused by potentially pathogenic microorganisms. These studies have also revealed key insights into the mechanisms behind the actions exerted by this probiotic. In this manuscript, we aim to provide a concise overview of the effects of *L. fermentum* administration on broiler chicken health and performance.

## 1. Introduction

In animal farming, antibiotics have been utilized not only for prophylaxis purposes but also for growth promotion, notwithstanding the forthcoming health threat associated with resistance [1,2,3]. The use of antibiotics as growth promoters has been forbidden in the U.S. and European Union [4,5], although this practice is still common in other regions, principally in rural areas that lack efficient administrative systems and legislative measures to curb drug misuse [6,7]. As a result, considerable attention has been drawn to the investigation of alternatives (e.g., probiotics) to replace the use of antibiotics for feed enrichment in animal production [8,9,10].

Probiotics have been defined by the Food and Agriculture Organization (FAO) and World Health Organization (WHO) as “live microorganisms that, when consumed in adequate amounts, confer a health effect on the host” [11]. However, an expert panel later reworked the definition to be utilized as follows: “products that deliver live microorganisms with a suitable viable count of well-defined strains with a reasonable expectation of delivering benefits for the wellbeing of the host” [12]. The most common probiotic microorganisms are bifidobacteria and lactic acid bacteria, although others are commonly recognized, including *Enterococcus*, *Lactococcus*, *Streptococcus*, *Propionibacterium*, and the yeast *Saccharomyces* [13]. Lactobacilli are acknowledged as the main contributors to intestinal homeostasis in humans and other animals [14,15,16,17], and the genus *Lactobacillus* is certainly the most studied of the lactic acid bacteria group, with more than 200 species described [18].

The beneficial effects exerted by probiotics are associated with various characteristics. First, these microorganisms are capable of adhering to and activating epithelial cells via surface proteins and other membrane-associated molecules (e.g., lipoteichoic acid (LTA) and exopolysaccharides (EPS)) [19,20]. This interaction not only enhances intestinal barrier function but also improves the balance of intestinal microbiota, thus preventing dysbiosis and epithelial dysfunction [21,22]. Additionally, interaction with the gastrointestinal tract allows for the competitive exclusion of pathogens [23,24]. Second, the secretion of compounds with bacteriostatic activity, such as organic acids and antimicrobial peptides, helps inhibit the growth of potentially harmful bacteria [25,26]. Finally, probiotics modulate the immune response of the host by interacting with key receptors that prompt the transcription of cytokines, which ultimately influence the production of immunoglobulins [27]. Despite the efficacy of probiotic administration, the extent of these general actions appears to be not only species- or strain-specific but also dose-dependent [28,29,30,31]. Thus, improving our knowledge of the benefits and the underlying mechanisms behind them is crucial for properly characterizing strains aimed at being used in animal production. As the broiler industry detaches from the utilization of antibiotics, novel strategies for prophylaxis and performance enhancement have been developed. For instance, probiotics and prebiotics, as well as plants and algae-derived products, have proven convenient for ameliorating intestinal and immune parameters, which ultimately were observed to enhance animal performance [32,33,34,35,36].

*L. fermentum*, in particular, is a well-characterized and highly recognized probiotic that is capable of adhering to epithelial cells, synthesizing antimicrobial compounds, and activating receptors that trigger the expression of immune-associated genes. Hence, it has been recently studied in land and marine animals. In pigs, for instance, the application of the probiotic, alone or in combination with other bacteria, enhances growth performance, digestibility, gut environment, and health status. *L. fermentum* treatment positively modulated the intestinal microbiota while alleviating inflammation in weaned pigs. Moreover, administration of a diet fermented with a probiotic mixture, including *L. fermentum* K9-2, reduced the load of intestinal pathogens such as *Escherichia coli* and *Clostridium perfringens* [37,38,39]. The application of this species has also yielded positive results in marine farming. Exposure to *L. fermentum* R3 Biocenol™ (CCM 8675) improved the mucosal health of Atlantic salmon [40], while supplementation of *L. fermentum* URLP18 and *L. fermentum* PTCC 1638 did not only improve growth conditions by modulating the immune as well as the antioxidant response but also relieved the pathogenic effects of *Aeromonas hydrophila* [41,42]. Another strain, *L. fermentum* 1744 (ATCC 14931), proved convenient for preventing the potential accumulation of heavy metals in rainbow trout [43]. This has also been observed in shrimp fed a diet enriched with *L. fermentum* GR-3, in which arsenic levels were diminished by the probiotic [44]. In general, the aforementioned benefits have also been observed after the inclusion of *L. fermentum* strains into shrimp diets; namely, lactic acid bacteria did not only ameliorate growth performance and health status but also provided protection against *Vibrio parahaemolyticus* [45,46,47].

Many studies have also reported the effects of probiotic administration on different physiological parameters in birds [48,49]. In poultry husbandry, various strains of *L. fermentum* have proved useful for enhancing growth conditions, which have been associated with gut health, nutrition, and modulation of the immune response. The potential of *L. fermentum* to counteract the effects of harmful bacteria has also been reported. In this review, we aimed at summarizing the evidence of the benefits of *L. fermentum* use in broiler chickens.

## 2. Properties of *Limosilactobacillus fermentum*

*Limosilactobacillus fermentum* was formerly known as *Lactobacillus fermentum*, and the taxonomy of *Lactobacillaceae* was revisited based on different approaches, including genomics and proteomics [50,51,52]. The genus classification refers to the synthesis of exopolysaccharides (limosus—slimy) [50]. The rod-shaped *L. fermentum* is recognized as a gram-positive, non-sporulating, catalase-negative, gas-producing facultatively anaerobic bacterium that is heterofermentative and capable of utilizing several carbohydrates, including arabinose, cellobiose, galactose, and maltose, among others [50,52,53,54]. Strains of *L. fermentum* are acknowledged as nomadic or free-living and occur spontaneously in different environments. They have been isolated not only from fermenting plant materials and fermented cereals but also from dairy products, sewage, manure, and the gastrointestinal tract and feces of birds, pigs, and humans [50,55,56,57,58]. Indeed, *L. fermentum*, as well as other lactobacilli, remain physiologically active in the gastrointestinal tract, with the potential to influence host physiology [55]. *L. fermentum* strains are known for exerting beneficial effects on human health [59,60,61,62,63]. This species is recognized as safe and is included in the official lists of European, American, and Chinese food safety authorities [64,65,66]. It has also been used for developing commercially available dietary supplements [61,67]. Selected strains have demonstrated particular probiotic characteristics that render them beneficial for the host (Table 1).

Once inside the host, probiotic bacteria are exposed to different types of stress, including low pH and elevated concentrations of bile salts. *L. fermentum* strains have evidenced high viability when encountering such conditions [57,68,69]; additionally, *L. fermentum* not only exhibits strong surface hydrophobicity but also high autoaggregation capacity; these characteristics have been associated with a facilitated interaction between bacterial and intestinal epithelial cells [53,70]. In general, lactobacilli are capable of adhering to intestinal mucosa [53,71,72]; this process is mainly mediated by adhesion proteins (e.g., binding proteins, sortases), but other molecules are also involved (e.g., LTA, LPS, PG) [53,73]. Particularly in *L. fermentum*, mucin- and fibronectin-binding proteins (Mub and Fbp, respectively), along with sortases, have been determined, with upregulation of *mub*, *fbp*, and *sor* observed in the presence of mucin, bile, and pancreatin [71,72]. Lipoteichoic acids have also been held responsible for the adhesion capabilities of some strains, along with other factors, including electrostatic interactions or passive forces [74,75]. Adherence of these molecules has proved beneficial for maintaining the integrity of the gut barrier; for instance, the LPS of *L. fermentum* CECT5716 increased the production of mucins in model intestinal cells [76]. This interaction permits the competitive exclusion of potential pathogens such as *Helicobacter pylori*, *Campylobacter jejuni*, and *Staphylococcus aureus* [77,78,79]. Pathogen clearance is enhanced by the capacity of *L. fermentum* strains to produce a variety of antimicrobial compounds, commonly known as bacteriocins. These ribosomally synthesized peptides are capable of disturbing the membrane or inducing cell wall degradation, although the mode of action of certain peptides remains unknown [79,80,81]. Various strains have been linked to these antimicrobial compounds (e.g., fermencin SD11, LF-BZ532, LBM97-1, LBM97-4, and LBM97-5), which have shown activity against gram-positive and gram-negative bacteria such as pathogenic *E. coli*, *Salmonella* spp., *S. aureus*, or *Listeria* spp. [82,83,84,85]. Also, other secondary metabolites (e.g., lactic and organic acids, hydrogen peroxide) contribute to the overall antibacterial activity of *L. fermentum* [78,86,87]. Bacterial infections can influence the concentration of reactive oxygen species/reactive nitrogen species (ROS/RNS) with the potential to induce pathological effects [88,89]. Some *L. fermentum* strains possess the entire glutathione-associated complex, which has made them attractive as potential modulators of oxidative stress [90,91,92]. This active redox tripeptide can reduce oxidative agents directly or indirectly as a cofactor of a group of enzymes involved in eliminating electrophilic compounds [93,94]. Moreover, the presence of *L. fermentum* is known to activate receptors that ultimately favor the transcription of antioxidant genes, which lessens oxidative stress [95].

**Table 1:** Probiotic properties of *L. fermentum* strains.

Strain	Origin	Functional Properties	References
*L. fermentum* YLF016	Yak gut	High survival rate in the gut; strong adherence to intestinal cells; antibacterial and antioxidant effects; non-hemolytic activity	[53]
*L. fermentum* PC-10	Poultry gut	Inhibition of *S.* Gallinarum growth	[56]
*L. fermentum* PG1	Poultry digesta	Adhesion to the epithelial cells; survival at low pH; tolerance to bile salts; antibacterial activity	[57]
*L. fermentum* Y57	Artisanal yogurt	Reduction of hypercholesterolemia in rats	[62]
*L. fermentum* GR-3	Fermented food	Ameliorates human hyperuricemia via degrading and promoting excretion of uric acid	[63]
*L. fermentum* MBD93	–	Adhesion to gastrointestinal mucin; exclusion of enteropathogenic bacteria	[71]
*L. fermentum* 10	Human feces	Strong adhesion to * HT29 epithelial cells; high tolerance to bile salt; autoaggregation activity; reduction of *E. coli* adhesion; antibacterial and antioxidant activity	[75]
*L. fermentum* J23	Cheese	Antimicrobial activity of bacteriocin-containing fractions; growth inhibition of *E. coli*, *S. aureus*, *L. innocua*, and *S.* Typhimurium	[82]
*L. fermentum* SD11	Human oral cavity	Production of fermencin SD11; antibacterial activity against oral pathogens	[83]
*L. fermentum* BZ532	Cereal beverage	Production of bacteriocin LF-BZ532 with a broad antimicrobial spectrum, including anti-listerial and anti-pseudomonas activity	[84]
*L. fermentum* LBM97	Fermented vegetable	Production of bacteriocins LBM97-4 and LBM97-5 with antibacterial activity against *S. aureus* and *E. coli*	[85]
*L. fermentum* ME-3	Human feces	Complete glutathione system; protection against oxidative stress	[90]
*L. fermentum* JX306	Fermented vegetable	High scavenging activity of free and hydrogen radicals; improving glutathione peroxidase activity; effective inhibition of oxidative damage in liver and kidney	[92]
*L. fermentum* UCO-979C	Human gut	Inhibition of *H. pylori* growth and urease activity	[77,96]
*L. fermentum* DLBSA204	Human breast milk	Macrophages activation; induction of nitric oxide synthesis; virus inactivation; downregulation of pro-inflammatory cytokines	[97]
*L. fermentum* IM12	Human gut	Inhibition of NF-κB-STAT3 signaling pathway	[98]
*L. fermentum* AGR1487	Human oral cavity	Capacity to activate TLR signaling pathway, immunomodulatory effects	[99]
*L. fermentum* CECT5716	Human breast milk	High production of mucins; intestinal anti-inflammatory effects; immunomodulatory effects; alleviation of colitis-associated dysbiosis; glutathione-associated complex; mastitis prevention	[60,67,76,91,100,101]

* HT29: human intestinal epithelial cell line.

*L. fermentum* interacts with intestinal epithelial cells (IECs), macrophages, dendritic cells, and immune cells; this induces the expression of different cytokines that modulate T cell polarization [65]. Such interactions are, on the one hand, associated with LTA, LPS, or PG of bacteria and, on the other hand, with Toll-like receptors (TLR2 and TLR4) and nucleotide-binding oligomerization domain-containing proteins (NOD2) of the host. This triggers the recruitment of adaptor proteins (MyD88, NF-κB) that transduce the signal to the nucleus and modulate the expression of response genes (e.g., cytokines) [102]. In intestinal cells, *L. fermentum* UCO-979C decreased expression of TNF-α, IL-1β, IL-6, and MCP-1 in *H. pylori*-challenged cells, although a slight increase was observed when compared to control conditions [96]. Exposure to *L. fermentum* CECT5716 also modulated the expression of TNF-α, IL-1β, and IL-6 in CMT-93 cells, which are used as a model cell line of the intestine [76]. Furthermore, *L. fermentum* DLBSA204 did not only activate macrophages and induce the synthesis of nitric oxide linked to bacterial clearance, virus inactivation*,* and tumor cytotoxicity but also reduced the expression of IL-6 and IL-1β [97]. Other strains (UCO-979C, IM12) have also demonstrated the ability to alter the expression of cytokines and other signaling molecules in macrophages [96,98]. In dendritic cells, *L. fermentum* AGR1487 modulated transcription of IL-6, TNFα, IL-10, and IL-12, whereas *L. fermentum* CECT5716 could induce the expression of MHC class II and other costimulatory molecules (e.g., CD40, CD80) [99,100]. The latter strain, when incubated with peripheral blood mononuclear cells (PBMCs), induced the activation of NK and Treg cells along with the production of cytokines including IL-1β, IL-18, TNF-α, and IFN-γ. PBMCs are constituted of lymphocytes and monocytes and are utilized for screening molecules with immunomodulatory properties [101]. The use of these cells has also demonstrated that exposure to *L. fermentum* B633 suppressed the production of IL-13 while prompting the synthesis of IL-12 and IFN-γ [103].

## 3. Applications of *L. fermentum* in Broiler Chickens

Broiler chickens have been bred exclusively for meat consumption, and the efficiency of the industry has been linked to innovations in management practices, breeding, nutrition, and disease control. However, complications from intestinal infectious diseases have negatively influenced production parameters, so antibiotics along with vaccines have extensively contributed to the efficiency of large-scale commercialization [104,105]. As the industry is detaching from the use of antibiotics for prophylaxis and performance, novel schemes have emerged for pathogen control and body weight enhancement, including probiotics, prebiotics, plants and algae, organic acids, bacteriophages, and essential oils [32,33,34,35,36,106]. Probiotics, in general, modulate key physiological characteristics that ultimately ameliorate animal development [48,49]. Strains of *L. fermentum*, in particular, have proven convenient for augmenting growth parameters, which has been related to their abilities to improve gut health by regulating architecture, epithelial integrity, microbial diversity, and inflammation. Moreover, these strains have been employed to antagonize the effects of potentially harmful bacteria such as *Campylobacter*, *Salmonella*, *Clostridium*, and *Pasteurella* (Table 2).

### 3.1. Gut Health, Microbiota, and Homeostasis

The gut ecosystem is acknowledged as a complex environment involving different constituents. The gut epithelium not only acts as a barrier against invading microorganisms and their toxins but also plays a fundamental role in host immunity and nutrient acquisition [125,126]. Intestinal epithelial as well as immune-associated cells are of prime importance; the metabolism of these cells could be modulated by various factors including age, housing, gender, or diet [127,128]. Furthermore, the development of a stable microbiota is known to stimulate the immune system and prevent enteric diseases [129,130,131]. A suspension of *L. fermentum* Biocenol CCM 7514 (1 × 10^9^ CFU/0.2 mL), administered orally during the first week of growth, augmented villus height in the small intestine in 8-day-old and 11-day-old chicks. The probiotic ultimately improved the villus-height-to-crypt-depth (VH:CD) ratio in the duodenum and ileum; a positive correlation between such conditions and the animal body weight was also determined [107]. This strain has also improved the aforementioned parameters in duodenal and jejunal sections of 15-day-old chicks; however, in this case, the number of goblet cells was determined and proved to be higher in animals exposed to the probiotic than in untreated ones, although no differences were observed regarding the expression of *muc2* [108]. On the contrary, in jejunal and ileal sections of 21-day-old chicks inoculated with *L. fermentum* 1.2029 (1 × 10^8^ CFU/0.5 mL), expression of this gene was higher than that of untreated birds. Nonetheless, an overall increment of goblet cell density was only evidenced in the jejunum [111].

Dietary supplementation of *L. fermentum* KGL4 (1 × 10^8^ CFU/mL) during the starter phase did not alter intestinal architecture; although a decrease in coliform and enterococci counts was reported, this was accompanied by a proliferation of lactobacilli. An overall increase in animal body weight was observed in probiotic-treated animals [114]. Likewise, dietary administration of *L. fermentum* NKN51 (1 × 10^7^ CFU/gM) for a period of 28 days reduced the total count of cecal *E. coli* while augmenting those of lactobacilli. In jejunal sections, this strain improved villus height, villus width, VH:CD ratio, and surface area; feed conversion ratio and body weight were also ameliorated [115]. Moreover, birds fed a diet containing *L. fermentum* 1.2133 (2.5 × 10^8^ CFU) showed larger numbers of lactic acid bacteria than control animals in the ileum and cecum; in the latter, a reduction in *Salmonella* counts was also registered [116]. Finally, *L. fermentum* has been used to develop multi-strain probiotics with potential applications in broilers. For instance, this species, along with *L. plantarum*, *Pediococcus acidilactici*, *Enterococcus faecium*, and *Saccharomyces cerevisiae*, has been mixed at equal ratios and added to the diet at a dose of 1 × 10^8^ CFU/kG between the third and 21st days. Incorporation of this mixture into the diet did not only reduce enterobacteria counts but also augmented the number of lactobacilli in both the ileal and cecal contents of 28-day-old chicks. Exposure to the probiotic also improved body weight and the feed conversion ratio [117]. Furthermore, a rapeseed meal fermented with a mixture of probiotics, including *L. fermentum* CICC 20176 and *L. fermentum* CGMCC 0843, improved the VH:CD ratio in the jejunum and ileum of 21- and 42-day-old chicks; no differences were found regarding animal performance [118,119].

Nutrition is crucial not only for sustaining the prooxidant-antioxidant balance but also for regulating fat metabolic function [132,133]. Reactive oxygen or nitrogen species can modulate primary immune defense, albeit prolonged exposure leads to a disruption of the oxidant/antioxidant network; this imbalance ultimately results in an acceleration of pathological inflammation [134,135]. The inclusion of *L. fermentum* CCM 7158 (1 × 10^9^ CFU) in drinking water reduced the total antioxidant status in 42-day-old broiler chickens, although it influenced neither bilirubin nor albumin levels. Its administration, however, reduced the content of serum triglycerides. This has also been observed in chickens (42 days old) fed a diet enriched with *L. fermentum* KGL4 (1 × 10^8^ CFU/mL); furthermore, the probiotic reduced LDL content while augmenting levels of HDL. In both cases, an increment in body weight was observed in probiotic-treated animals [114,120]. Similarly, *L. fermentum* CIP 102980 (1 × 10^7^ CFU/mL) improved growth performance and feed conversion ratio in 36-day-old birds [121].

### 3.2. Modulation of Immune Reaction

Strains of *L. fermentum* are recognized for their immunomodulatory properties, as they are able to interact with immune cells and either suppress or stimulate the production of various inflammatory cytokines [136,137,138]. Oral administration of *L. fermentum* Biocenol CCM 7514 (1 × 10^9^ CFU/0.2 mL) during the first week of growth did not only induce expression of anti-inflammatory cytokines (IL-13, IL-4), but also reduced transcription of pro-inflammatory factors in the cecum of one-week-old chickens, including IL-15, IL-16, IL-17RA, LIF, IL-6RA, and CXCL-12 [107,109,110]. This treatment also increased the percentages of lamina propria IgM plasma cells and intraepithelial CD8 cells [109]. The latter were also augmented in the jejunum of 21- and 42-day-old chickens when a probiotic product was added to the basal diet; this product contained 1× 10^7^ CFU/g of *L. fermentum* JS and 2 × 10^6^ CFU/g of *S. cerevisiae*. The percentages of intraepithelial CD4 and CD3 cells were also enhanced, and overexpression of TLR2 and TLR4 was registered [122]. Additionally, a mixture of probiotics, containing approximately 5 log CFU/mL of *L. fermentum* CICC 20176 and *Bacillus subtilis* (1:1), was used to ferment a meal based on rapeseed; dietary administration of this mixture improved the concentration of serum IgG and IgM in 21-day-old chickens [118].

### 3.3. Antagonism against Potentially Harmful Bacteria

The ability of *L. fermentum* to antagonize a variety of dangerous bacteria is not only associated with competitive exclusion but also with the secretion of bacteriocins and secondary metabolites that contribute to the overall antimicrobial activity [77,78,82,83,84,85]. Moreover, stimulation of the immune system by *L. fermentum* could prime the host’s response to potential infections [96,98]. For example, the use of *L. fermentum* Biocenol CCM 7514 could prime the immune response during *Campylobacter* spp. infections. *Campylobacter* has been traditionally regarded as commensal in birds, although it has been reported that its presence induces the expression of pro-inflammatory cytokines, which may lead to intestinal damage and ultimately to weight loss [139,140]. Inoculation with the probiotic (1 × 10^9^ CFU/0.2 mL) during the first week of growth enhanced the immune response in 8-day-old challenged chicks. In cecal sections, the percentage of CD8 and IgA plasma cells in the epithelium and lamina propria was augmented compared to *C. coli*-infected animals; furthermore, a downregulation of inflammatory cytokines (e.g., IL-15 and IL-16) was also observed [109]. A similar cecal response has been registered in the context of a *C. jejuni* infection; early treatment with the aforementioned strain (1 × 10^9^ CFU/0.2 mL) modulated the expression of inflammatory cytokines, including IL-1β, IL-17, and IL-15, in 8-day-old challenged chicks. Moreover, in these animals, *C. jejuni* invasion reduced the height of villi in the duodenum, jejunum, and ileum; in the latter section, crypt depth was also affected. Application of *L. fermentum* Biocenol CCM 7514 did not only prevent these effects but actually ameliorated intestinal architecture, even when compared to untreated animals [107,110].

Different serovars of *Salmonella* are capable of eliciting intestinal mucosal damage in broiler chickens [141,142]. The beneficial effects exerted by *L. fermentum* Biocenol CCM 7514 regarding gut health have also been evidenced in chickens challenged with *S.* Infantis. Infection with this serovar reduced the VH:CD ratio in the small intestine of 15-day-old birds. Early probiotic treatment (1 × 10^9^ CFU/0.2 mL) did not only relieve the observed impairments but also improved the calculated ratios when compared to basal levels. In animals previously exposed to the probiotic, the presence of *S.* Infantis increased the surface of villi and augmented the number of goblet cells in the small intestine compared to control conditions. Finally, higher IgM serum levels were also reported in the co-exposure group than in untreated birds [108]. Infection with *S.* Pullorum also affected intestinal homeostasis in 15-day-old chicks. First, the pathogen decreased total anaerobic bacteria while increasing the number of total aerobic bacteria in the ileum and cecum; these outcomes were relieved by animal exposure to *L. fermentum* 1.2133 (2.5 × 10^8^ CFU). In particular, probiotic administration reduced the presence of *Salmonella* in challenged animals. Second, *S.* Pullorum infection triggered lesions in duodenal villi, evidencing accumulation of erythrocytes and autolysis; the latter was also observed in ileal goblet cells. Previous inoculation with the probiotic relieved these conditions, as few erythrocytes were found in villi and injuries were local and fewer in number [116]. Similarly, *S*. Enteritidis negatively affected intestinal homeostasis, as it elicited hemorrhagic lesions and the expression of inflammatory cytokines (IL-1β and LITAF) in the cecal tonsils of 11-day-old chickens. These effects were lessened by oral inoculation of a mixture called *Lactobacilli*-based probiotic, containing *L. acidophilus*, *L. reuteri*, *L. salivarius*, and *L. fermentum* (1 × 10^5^ CFU). Ingestion of the mixture proved to increase the percentage of macrophages and CD4 T cells, which was not observed when birds were only infected with *S*. Enteritidis [123].

*C. perfringens* is associated with intestinal barrier damage, unstable intestinal microbiota, and reduced immunity in birds [143,144]. *L. fermentum* strains have also been shown to be beneficial in diminishing pathogenic outcomes induced by these bacteria, regardless if the probiotic was supplemented orally or in the diet. First, oral administration of *L. fermentum* 1.2029 (1 × 10^8^ CFU/mL) demonstrated protection against the negative effects caused by *C. perfringens* in the ileum of 28-day-old animals. Infection prompted the upregulation of inflammatory factors, such as IFN-γ and TLR2, and the downregulation of IL-10. The latter was upregulated in the presence of the probiotic, whereas the former two were downregulated. In addition, the pathogen induced-hyperplasia of the lamina propria*,* along with lymphocyte infiltration and crypt structure deterioration. Again, the lesions derived from infection were not detected in birds previously exposed to the probiotic strain [112]. Second, the incorporation of *L. fermentum* (1 × 10^9^ CFU/g) into the basal diet relieved the intestinal damage elicited by *C. perfringens* in 13-day-old chickens, which involved a decrease in VH:CD ratio in the duodenum, jejunum*,* and ileum as well as a downregulation of key factors including ZO-1, Mucin-2, and Occludin in the jejunum. Previous exposure of infected animals to probiotic treatment induced even better conditions than those registered in untreated birds [124]. Likewise, *C. perfringens* inoculation stimulated the expression of the pleiotropic and potentially inflammatory cytokine TGF-β4 in the jejunum; such expression levels were reduced by dietary administration of *L. fermentum* 1.2029 (1 × 10^9^ CFU/kG) in 21-day-old animals. However, treatment with the probiotic also increased transcription of cytokines such as IL-1β, IFN-γ, IL-17, and TGF-β4 in older chicks (28-day-old); this has been linked to the inhibitory and stimulatory effects of the probiotic in both the acute and recovery phases of infection [113]. Finally, *P. multocida* causes the contagious disease known as “avian cholera”, which is linked to high morbidity and mortality [145]. Infection by *P. multocida* did not only alter the ileal and cecal microbiota but also reduced body weight and increased mortality rates in 28-day-old chickens. A mix of probiotics, including *L. fermentum*, was supplemented in the feed (1 × 10^8^ CFU/kG). Challenged animals exposed to the enriched diet showed no evidence of *P. multocida* effects on the intestine; body weight loss and mortality rates were also attenuated. In general, previous exposure to the probiotic reduced intestinal enterobacteria counts while augmenting the total number of lactic acid bacteria. Furthermore, the probiotic mixture reduced cholesterol and glucose while eliciting the production of lymphocytes and upregulating the expression of anti-inflammatory genes in the cecal mucosa [117].

**Figure 1:**
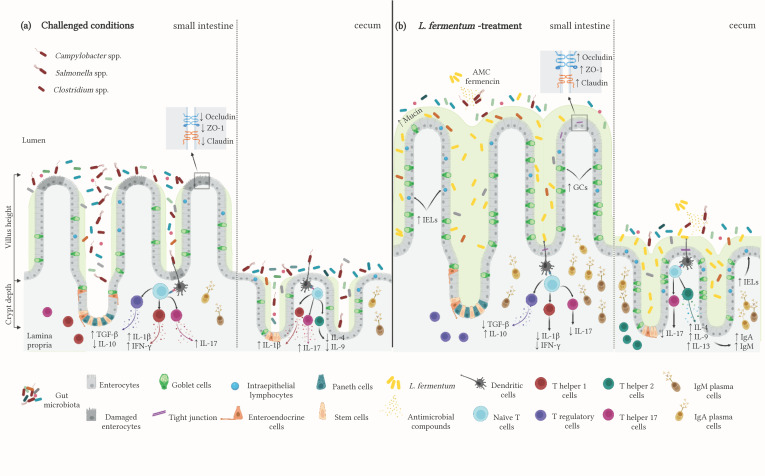
*L. fermentum* interacts with intestinal epithelial cells and gut microbiota. (**a**) Impairment of villi and crypt architecture, along with intestinal lesions and limited mucin production, has been observed in chickens challenged with *Campylobacter*, *Salmonella*, or *Clostridium* spp. Moreover, these conditions alter the composition of the intestinal microbiota and prompt the production of inflammatory factors. (**b**) Various strains of *L. fermentum* have shown the ability not only to attenuate these responses but also to improve the overall intestinal environment. *L. fermentum* is known for synthesizing antimicrobial compounds (AMC) (e.g., fermencin) and for competitively excluding other bacteria, thus supporting the development of a stable microbiota and reducing the effects of potentially harmful microorganisms. Indeed, *L. fermentum* treatment proved useful for ameliorating the VH:CD ratio and also for augmenting the number of goblet cells. Probiotic exposure also induced the downregulation of pro-inflammatory factors while upregulating the Th2 immune response. For references, please see Table 2. Created with BioRender.com (accessed on 14 June 2023-Agreement N° IS25JSZ31U). Figure symbols: ↑ increment; ↓ reduction.

Results from animal trials involving *L. fermentum* strains have demonstrated the beneficial effects of this probiotic on intestinal health and growth performance. These outcomes have also evidenced the protective effects of *L. fermentum* against potential pathological conditions induced by other bacteria, as it can adhere to the epithelium and secrete antimicrobial compounds. Moreover, treatment with these lactic acid bacteria improves intestinal health, namely gut architecture as well as the immune response (Figure 1). Despite the relevance of current research, further studies must be conducted to ensure the safety and efficiency of these strains, especially regarding possible side effects.

## 4. Conclusions

The in vivo studies summarized here exhibit the beneficial effects of *L. fermentum* administration on broiler chicken physiology and growth, especially with regards to gut health, nutrition, and modulation of the immune response. Furthermore, this species has demonstrated the potential for antagonizing the negative effects exerted by potentially pathogenic bacteria. In particular, strains of *L. fermentum* have proven beneficial for ameliorating conditions in the small intestine, including VH:CD ratio, microbial composition, integrity of the epithelium, and inflammation. Broiler chickens are bred for meat, and the productivity of the industry has been associated with management, breeding, and disease control practices that normally employ antibiotics for both prophylaxis and performance. However, due to the public concerns raised by the use of antibiotics in animal husbandry, many countries have banned their use as growth promoters. Thus, alternatives must be designed not only to maintain production performance but also to curb the effects of infectious diseases. Probiotics have been established as a potential strategy for preventing the disruption of the gut microbiota and preserving intestinal homeostasis. They represent a possible feed additive that may, or may not, have an influence on profitability; however, in the absence of antibiotics, these species definitely represent an important option for supporting animal growth and providing protection against invading pathogens. A variety of *L. fermentum* strains, administered orally, dietary, or in drinking water, have proved advantageous for improving such conditions in broiler chickens. Further research, however, should not only focus on determining the effects of probiotics on animal physiological conditions but also on deciphering the mechanisms behind their action, which might lead to the discovery of novel potential therapeutic targets. Undoubtedly, the evidence gathered so far demonstrates that *L. fermentum* should be considered as a potential ingredient when developing nutritional supplements aimed not only at improving growth conditions but also at preventing and treating infectious diseases.

## Figures and Tables

**Table 2:** Effects of *L. fermentum* application in broiler chickens.

Strain	Dose	Administration	Main Results	References
*L. fermentum* Biocenol CCM 7514	1 × 10^9^ CFU/0.2 mL	Orally	↑ VH and ↑ VH:CD ratio in the small intestine; ↑ GC count in the duodenum and jejunum; positive correlation between gut architecture and BW in early stages ↑ mRNA expression of IL-4, IL-18, IL-13; ↓ mRNA expression of IL-15, IL-16, IL-17RA, IL-9, IL-6RA and CXCL-12; ↑ percentages of IgM and CD8 cells in the cecum of young chickens Antagonistic effects against *C. jejuni*, *C. coli*, and *S.* Infantis; attenuation of intestinal impairments and regulation of cecal inflammatory response	[107,108,109,110]
*L. fermentum* 1.2029	1 × 10^8^ CFU/0.5 mL	Orogastrically	↑ jejunal GC density; ↑ mRNA expression of *muc2* in the jejunum and ileum of 21-d-old chickens	[111]
	1 × 10^8^ CFU/mL	Orally	Lessening of *C. perfringens*-induced conditions; intestinal necrotic lesions not observed after treatment; ↑ mRNA expression of IL-10; ↓ mRNA expression of IFN-γ and TLR2 in 28-d-old chickens	[112]
	1 × 10^9^ CFU/kG	Dietary	Modulation of *C. perfringens*-stimulated expression of pro-inflammatory cytokines in the jejunum in 28-d-old chickens	[113]
*L. fermentum* KGL4 and *L. plantarum* KGL3A complex	1 × 10^8^ CFU/mL	Dietary	↓ fecal coliform and enterococci count; ↑ fecal lactobacilli count during initial growth phase; well-organized intestinal epithelial lining and villi structure; ↑ BW; ↓ LDL and ↑ HDL content in serum of 42-d-old chickens	[114]
*L. fermentum* NKN51	1 × 10^7^ CFU/gM	Dietary	↓ total count of *E. coli*; ↑ count of lactobacilli; ↑ VH, VW, VH:CD ratio and surface area in the jejunum; ↑ BW and ↓ FCR of 28-d-old chickens	[115]
*L. fermentum* 1.2133	2.5 × 10^8^ CFU	Dietary	↑ number of lactobacilli in the ileum and cecum; ↓ *Salmonella* counts in the cecum of 15-d-old chickens Lessening of intestinal lesions inflicted by *S.* Pullorum	[116]
*L. fermentum* (strain unspecified)	1 × 10^8^ CFU/kG	^1^ Dietary	↓ enterobacteria counts, ↑ lactobacilli counts in ileum and cecum; ↑ BW and ↓ FCR of 28-d-old chickens Reduced effects of *P. multocida* on intestinal microbiota; regulation of anti-inflammatory genes	[117]
*L. fermentum* CICC 20176	approx. 5 log CFU/mL	^2^ RSM fermentation	↑ VH:CD ratio in the jejunum; ↑ concentration of serum IgG and IgM; no differences in growth performance of 21- and 42-d-old chickens	[118]
*L. fermentum* CGMCC 0843	approx. 5 log CFU/mL	^3^ RSM fermentation	↑ percentages of dry matter digestibility in 42-d-old chickens; ↑ VH:CD ratio in the jejunum and ileum of 21- and 42-d-old chickens; ↑ lactobacilli count in the ceca and colon of 21- and 42-d-old chickens	[119]
*L. fermentum* CCM 7158	1 × 10^9^ CFU	In drinking water	↓ total antioxidant status; ↓ content of serum triglycerides; ↑ BW in 42-d-old chickens	[120]
*L. fermentum* CIP 102980	1 × 10^7^ CFU/mL	Intragastrically	↑ BW and ↓ FCR in 36-d-old chickens	[121]
*L. fermentum* JS and *S. cerevisiae* product	1 × 10^7^ CFU/g	Dietary	↑ percentages of CD3, CD4, CD8 cells and ↑ mRNA expression of TLR2 and TLR4 in the jejunum of 21- and 42-d-old chickens; ↑ BW, ↓ FCR ratio during starter period	[122]
*L. fermentum* (strain unspecified)	1 × 10^5^ CFU	^4^ Orally	Protective effects against *S.* Enteritidis infection; ↑ percentages of macrophages and CD4 cells; minimized lesions in the cecal tonsils in 11-d-old chickens	[123]
*L. fermentum* (strain unspecified)	1 × 10^9^ CFU/g	Dietary	*C. perfringens*-induced downregulation of ZO-1, Mucin-2, and Occludin in the jejunum of 13-d-old chickens relieved by probiotic administration	[124]

^1^ Administrated in combination with *L. plantarum*, *P. acidilactici*, *E. faecium*, and *S. cerevisiae*; ^2^ in combination with *B. subtilis*; ^3^ in combination with *E. faecium*, *S. cerevisae*, and *B. subtilis*; ^4^ in combination with *L. acidophilus*, *L. reuteri*, and *L. salivarius*. VH: villus height; VW: villus width; GC: goblet cell; BW: body weight; FCR: feed conversion ratio; LDL: low-density lipoprotein; HDL: high-density lipoprotein; RSM: rapeseed meal. Table symbols: ↑ increment; ↓ reduction.

## Data Availability

Not applicable.
